# Developing models of how cognitive improvements change functioning: Mediation, moderation and moderated mediation

**DOI:** 10.1016/j.schres.2012.03.020

**Published:** 2012-06

**Authors:** Til Wykes, Clare Reeder, Vyv Huddy, Rumina Taylor, Helen Wood, Natalia Ghirasim, Dimitrios Kontis, Sabine Landau

**Affiliations:** a King's College London, Institute of Psychiatry, London SE5 8AF, United Kingdom; b Salomans Centre for Applied Social & Psychological Development Tunbridge Wells, United Kingdom; c Department of Psychiatry, University of Medicine & Pharmacy “Iuliu Hatieganu”, Romania; d 1st Psychiatric Department, Psychiatric Hospital of Attica, Athens, Greece

**Keywords:** Schizophrenia, Rehabilitation, Cognitive remediation therapy, Functioning

## Abstract

**Background:**

Cognitive remediation (CRT) affects functioning but the extent and type of cognitive improvements necessary are unknown.

**Aim:**

To develop and test models of how cognitive improvement transfers to work behaviour using the data from a current service.

**Method:**

Participants (N49) with a support worker and a paid or voluntary job were offered CRT in a Phase 2 single group design with three assessments: baseline, post therapy and follow-up. Working memory, cognitive flexibility, planning and work outcomes were assessed.

**Results:**

Three models were tested (*mediation* — cognitive improvements drive functioning improvement; *moderation* — post treatment cognitive level affects the impact of CRT on functioning; *moderated mediation* — cognition drives functioning improvements only after a certain level is achieved). There was evidence of *mediation* (planning improvement associated with improved work quality). There was no evidence that cognitive flexibility (total Wisconsin Card Sorting Test errors) and working memory (Wechsler Adult Intelligence Scale III digit span) mediated work functioning despite significant effects. There was some evidence of *moderated mediation* for planning improvement if participants had poorer memory and/or made fewer WCST errors. The total CRT effect on work quality was d = 0.55, but the indirect (planning-mediated CRT effect) was d = 0.082

**Conclusion:**

Planning improvements led to better work quality but only accounted for a small proportion of the total effect on work outcome. Other specific and non-specific effects of CRT and the work programme are likely to account for some of the remaining effect. This is the first time complex models have been tested and future Phase 3 studies need to further test mediation and moderated mediation models.

## 1 Introduction

A recent meta-analysis confirmed that there is a small to moderate effect of cognitive remediation (CRT) on global cognition even after the effect of moderators and mediators, including methodology, had been taken into account (Wykes et al., 2011). Cognition both directly and indirectly (through negative symptoms) impacts interpersonal behaviour, work, community and household functioning (Wykes et al., 2007a; Fiszdon et al., 2008) so it is expected that these CRT derived cognitive improvements should carryover to improved function — a mediation model.

People who receive CRT do improve functioning, and this has been attributed to cognitive improvements (Spaulding et al., 1999; Wykes et al., 1999, 2003, 2007c, 2011; Reeder et al., 2004; 2006). However, there is scant direct evidence for this mediation model or for any other model of how therapy-induced cognition improvement impacts functioning. The absence of such models is currently hampering the development of cognition enhancement including drugs and CRT.

In terms of extant evidence, Fiszdon et al. (2008) showed that memory and executive performance changes predicted improvements in quality of life but the variables acted in opposite directions — memory improvements and executive decrements. Other studies have demonstrated a positive impact of both executive and memory improvements on social functioning (Wykes et al., 2007a) and social skills (Spaulding et al., 1999). Reeder et al. (2004, 2006) showed, in secondary analyses of several studies (Wykes et al., 1999, 2003, 2007b), that social functioning improvement was affected by a specific cognitive factor — responding to external feedback. This was despite other cognitive factors showing strong cross-sectional and longitudinal relationships with social functioning. This result has been independently replicated (Penades et al., 2010). Cognitive improvements cannot therefore be assumed to have an impact solely because they improve over time and were initially cross-sectionally related to functional outcomes.

Cognitive level may play a part in boosting recovery. Good working memory was associated with social behaviour recovery following an admission whereas those with poor working memory changed little over a year (Smith et al., 2002); and better baseline cognition is related to greater functional improvements (Brekke et al., 2007). Bell et al. (2005) found that those whose memory was within normal limits following CRT earned the most money. Cognition level affecting outcome improvement implies a moderation model such that those who achieved above a threshold would be more likely to accomplish functional gains following CRT.

A further, more complex, model might even suggest that the benefit of improved cognition is only translated into improvements in functioning in those who have also reached a critical cognitive level in other cognitive functions. For instance, a drug may improve some cognitive domains but require CRT to develop specific cognitive change in other domains. This is known as moderated mediation.

Modelling has been absent as CRT researchers have jumped from Phase 1 to Phase 3 and 4 studies (Wykes and Huddy, 2009; Wykes, 2010; Wykes and Spaulding, 2011). Identifying what is related to cognition (an *indirect effect*) and to therapy factors (a *direct effect*) should lead to the most efficient therapy development as it will more clearly define the therapeutic targets important to functional improvement. Models also enable the identification of weak links in the causal chain of existing therapies which might be strengthened. This view is consistent with that expressed in the Medical Research Council and NIMH guidance on the evaluation of complex or multi-component interventions which stress the importance of exploring different mechanisms leading to the therapeutic effect (Craig et al., 2008). This paper does not test CRT efficacy but shows how Phase 2 data might be utilised to explore explanatory models of how CRT leads to improvements. This will generate hypotheses for confirmatory testing in future Phase 3 efficacy and effectiveness studies.

We concentrate on work outcomes as they have shown consistent CRT effects. Current work outcomes are crude and as work placements may be limited with less opportunity to increase hours worked or payment in the current economic climate, using these outcomes would restrict the identification of future benefits when employment opportunities are scarce. So the quality as well as the quantity of work is included as an outcome. The aims are:-

• to assess the extent of cognition (executive and memory) and work improvement following CRT.
• to explore whether there is evidence of cognition playing a mediation, moderation or moderated mediation role in the relationship between CRT and improved functioning.

## 2 Materials and methods

### 2.1 Design and procedure

The study is a longitudinal follow-up of a single group of participants with the main outcomes, work behaviour and cognitive performance, assessed three times: weeks 1, 13 (post-treatment) and 25 (follow-up). After the baseline assessment all participants received 12 weeks of CRT.

### 2.2 Participants and sampling

Participants were drawn from those being supported by a key worker for voluntary or paid employment or within the educational system. They also received CRT. The models were designed to be tested with those who had received at least half the number of CRT sessions (> 20 sessions). Participants also fulfilled the following inclusion criteria:

• DSM-IV diagnosis of schizophrenia or schizo-affective disorder verified by case note review.
• No evidence of head injury or organic disorder.
• No primary substance abuse.

Participants were sampled from community mental health teams covering a defined geographical area in sequence. Criteria were checked from a potential participant list from each team and following referral were approached for written informed consent.

NHS Research Ethics Committee number: 05/Q0706/84.

### 2.3 Measures recorded over time

#### 2.3.1 Cognition

(i) Working memory (*memory*): WAIS-III Digit Span Test total score (Digit Span; Wechsler, 1999).
(ii) Cognitive Flexibility (*flexibility*): Wisconsin Card Sorting Test total errors (WCST; Heaton et al., 1993) in order to capture all impairments and improvements (Li, 2004).
(iii) Executive function (*planning*): the Zoo Map test total profile score (0–3) from the Behavioural Assessment of the Dysexecutive Syndrome (BADS; Wilson et al., 1996) which assesses executive function on real-life tasks. Participants are presented with maps of a zoo and asked to devise and execute a plan to visit places without breaking certain rules and within a time limit.

#### 2.3.2 Work

(i) The number of hours in structured occupation (work, education).
(ii) Work Behaviour Inventory Total Score (WBI; Bryson et al., 1997) examines behaviour in the workplace. It was completed by the research worker and has been used previously to assess CRT outcome (Kern et al., 2009). WBI work quality sub-score reflects only quality and not other factors improving during therapy.

#### 2.3.3 Baseline variables

(i) *Premorbid IQ*: Wechsler Test of Adult Reading (WTAR; Wechsler, 2001).
(ii) *Current IQ*: Short Wechsler Adult Intelligence Scale-III-UK using the WAIS-III Vocabulary and Block Design subtests (Jeyakumar et al., 2004).
(iii) *Other variables* were based on information from participants, case notes, mental health and employment workers. *Clinical* information included general functioning (Global Assessment of Functioning; (GAF; APA., 1994) and symptoms (HAD; Hamilton Depression Scale score Hamilton, 1980); PANSS: Positive and Negative Syndrome Scale (Kay et al., 1987); Rosenberg self-esteem — range 0–30 (Rosenberg, 1965). *Medication* was converted to anti-cholinergic load (Carnahan et al., 2006) and chlorpromazine equivalents (Woods, 2003; Taylor et al., 2009).

### 2.4 Therapy

CRT is a manual-based, individual psychological therapy (Delahunty et al., 2002) provided by trained graduate psychologists. Participants follow an individualized therapeutic protocol, which uses a variety of training techniques, which have led to improved cognitive performance in laboratory studies (Kern et al., 2002). It has three modules: flexibility, memory and planning, throughout which, cognitive processing strategies are explicitly taught (Wykes et al., 2007c). Hourly CRT sessions took place at least three days per week for 12 weeks, missed sessions were not repeated. Therapist, participant and participant's support worker met three times to discuss achievements and the strategies used in CRT to aid transfer of improvements to the workplace.

Therapist Fidelity was supported by clinical supervision and assessed by independent ratings of 50 session tapes on the CRT Fidelity Scale (Stenmark, 2006). All ratings confirmed that the key therapy components were administered to protocol.

### 2.6 Statistical analysis

#### 2.6.1 Sample size calculation

A sample size of 40 would give 80% power to detect a correlation of 0.41 or larger (moderate to large effect) (Wykes et al., 1992, 2011; Wykes, 1994) between work outcome change and cognition change, using a 2-sided test for zero correlation at the 5% significance level. Assuming a conservative drop-out rate of 20% this gave a final sample size requirement of 50 participants.

#### 2.6.2 Data analysis

This study is hypothesis and model generating with the objectives being (1) to assess change in cognition and work outcome and (2) assess relationships between cognitive change and functioning change at follow-up. Non-normally distributed variables were transformed. Any pathways likely to be fruitful areas for research were captured by adopting a 10% generous significance level with no adjustment for multiple testing. If pathways do not appear it is assumed that their effects are subtle or non-existent and therefore not important for future testing. Standardized effect sizes are provided and for changes over time this is Cohen's d or odds ratios, and for modelling these will be akin to partial correlation (beta) coefficients.

(1) Changes over time
*Changes over time* estimate the total effects of CRT and were analysed using mixed modelling with the outcomes recorded three times (dependent variables) and explanatory variables given by two dummy variables for time. Complete case analysis usually assumes that the data are “missing completely at random” (MCAR). To allow observed variables to drive missingness only “missing at random” (MAR) was assumed and predictors of loss to follow-up used as covariates in the analyses. Random intercepts for participants were included to allow for correlation between the repeated measures. The Zoo Map profile score is an integer score and was analysed using ordinal logistic regression with standard errors that are robust against correlations within clusters (subjects). To facilitate logistic mixed modelling this measure was dichotomised to indicate a positive planning score (0 if score = 0, 1 if score > 0).
(2) Modelling of relationships between changes
  The total CRT effect was partitioned to determine how (or if) cognitive change affects work. Regression models always included baseline variables that were predictive of missingness as covariates.
  To assess the potential for *mediation* (Model 1) the changes in work outcomes (time 3–time 1) were regressed on the changes in cognition (time 2–time1).
  Model 2 assumes that work outcome is *moderated* by the *level* of cognition (i.e. the change in work outcome depends on cognition level achieved following CRT). This relationship was assessed by a regression model in which work change was the dependent variable and the explanatory variable was the cognitive level achieved at time 2.
  Model 3 is *moderated mediation* where cognitive improvement affects work functioning in those that attain a critical cognitive level at the end of the therapy although this model can be extended to sub-groups defined in other ways e.g. by chronicity, medication type or symptom profile. If no step change was indicated graphically a median split was adopted. In these regression analyses work change is the dependent variable and the explanatory variables are (binary) cognitive level at time 2, change in cognition and their interaction. Mediation effects were tested in each of the sub-groups to assess the strength of the relationships for future study. The assumptions underlying the regressions for models 2 and 3 using diagnostic plots were examined and only those analyses where the assumptions were reasonable are reported.

## 3 Results

### 3.1 Participants

57 people consented and 8 were withdrawn before the study start (5 for an illness episode; 3 for personal reasons). The 49 people remaining attended: paid employment (5), volunteer employment (19), sheltered work (25). 43 participants received more than 20 CRT sessions. Of those not included (mean no. sessions = 8.3, range: 0–20), five people had other time commitments and one was admitted to hospital. There were no differences with those who completed a CRT course in terms of age, overall symptoms, hours worked when therapy began or duration in contact with services.

The study sample characteristics are shown in Table 1. 42 were treated with antipsychotic medication at study entry (36 atypical, 5 typical, 1 both). Average anti-cholinergic load was 2.7 (s.d. 2.2) and the mean chlorpromazine equivalents was 521 mg/day (s.d. 469).

### 3.2 Predictors of loss-to-follow up

Fewer structured occupation hours and higher baseline GAF score predicted missingness and were included as covariates in all analyses.

### 3.3 The total effect of CRT on cognition and work

Table 2 shows the results of the formal change analyses for cognition and work. All outcomes except structured occupation hours changed consistent with a CRT benefit and were significant for at least one post treatment time point. Standardized effect sizes were in the moderate range. Medication was not related to changes in any outcome.

### 3.4 The indirect CRT effects

#### 3.4.1 Model 1 — mediation

**Fig. 1:**
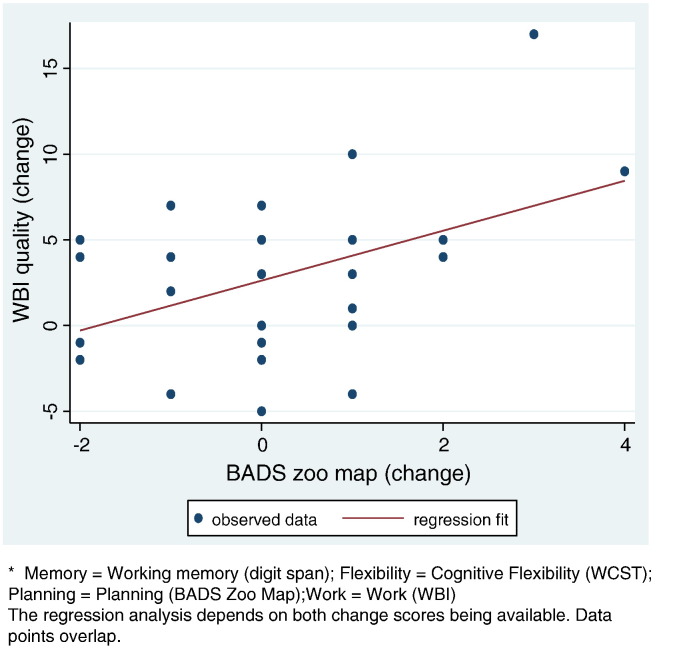
Effect of changes in Zoo Map score (time 2–time 1) on changes in work quality score (time 3–time 1).

The planning score change on WBI (both total and quality change) was significant. A one point improvement in Zoo Map score following CRT leads to 6.1 point improvement on WBI total score at follow-up (95% CI from − 0.6 to 12.7 points, p = 0.07, beta = 0.37) and a 1.58 point improvement on WBI work quality (CI from 0.28 to 2.88 points, p = 0.019, beta = 0.48, Fig. 1).

#### 3.4.2 Model 2 — moderation

There was no evidence of non-linear relationships and no linear relationship tested significant. There was no evidence that work changes depended on the level of cognition achieved post therapy.

#### 3.4.3 Model 3 — moderated mediation

This modelling investigated whether the memory or flexibility level might be necessary for improvements for planning to be transmitted into work changes. Since both cognitive flexibility and working memory are essential for efficient planning it is expected that those whose functioning might benefit most from planning improvements would be those having the fewest memory or flexibility problems.

Planning improvements may be transmitted into work quality improvement specifically for those with lower memory scores and increasingly for those with fewer WCST errors (Table 3). A detailed inspection of the data shows that two participants with the largest increase in planning and work quality have low memory scores and are therefore highly influential.

### 3.5 Process model for the total effect of CRT in supported work setting

**Fig. 2:**
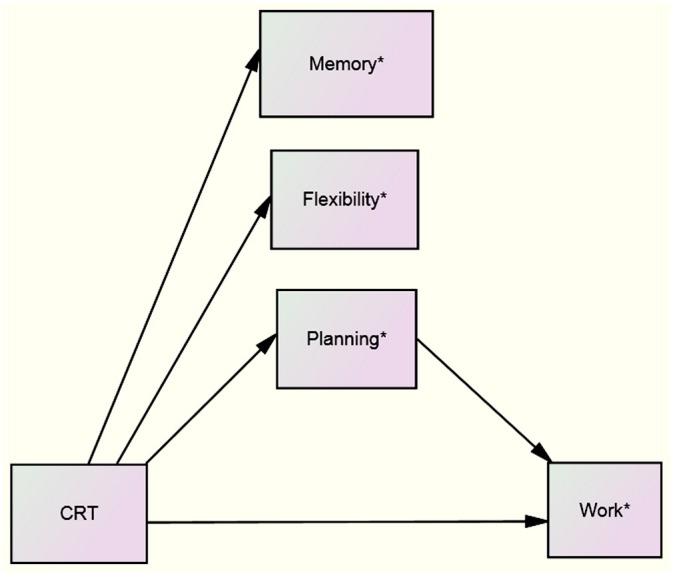
Proposed process model for CRT, cognition and work functioning in supported work setting. * Memory = working memory (digit span); flexibility = cognitive flexibility (WCST); planning = planning (BADS Zoo Map); work = work (WBI) The regression analysis depends on both change scores being available. Data points overlap.

The results suggest (Fig. 2):

• a potential CRT benefit on cognition (memory, flexibility and planning).
• a potential beneficial total effect of CRT on work functioning (WBI quality score).
• a potential association between improvements in planning and improvements in work functioning.

The standardized total effect of CRT on work quality was d = 0.55 (Table 2) and the indirect standardized effect of CRT on work quality (planning mediated CRT effect) was calculated as only d = 0.082.

## 4 Discussion

This Phase 2 study is not designed to test for CRT efficacy but to indicate signals for future investigation. There was a total CRT effect on all three cognitive measures and on work behaviour (total and quality scores) but unlike other studies (e.g. McGurk et al., 2009) the effects were not seen on structured occupation hours. The background here was a set of independent services with little integration of employers and limited opportunities in time and scope and although there was employment support (an inclusion criterion), the supporters were generally not trained in supported employment. However, even with this lack of a stable framework there was evidence that work behaviour improved.

### 4.1 Does cognition drive work functioning change?

The analyses suggest that one variable — planning/executive functioning was the only cognitive predictor of work improvement despite moderately sized and significant improvements in both memory and flexibility following CRT.

At the study outset it was assumed that work improvements might relate to a cognitive improvement threshold — a moderation model. In the Bell et al. (2005) study there was no significant interaction of level and work improvement for those with normalised memory scores. Rather it was in the comparison of remediation and no-remediation groups where there was a significant effect of cognitive level achieved. This suggests that it was remediation that was important in achieving a good outcome. However, the level response within the remediation group has not previously been investigated and it would have provided us with a guide on how to choose between cognitive remediation therapies that would have an impact on this area of functioning — i.e. those that produce larger changes in cognition. The data give no indication of this putative threshold.

There was some evidence of moderated mediation. The relationship between better flexibility and an improvement in work following a planning improvement is unsurprising as it is expected that those who have the fewest flexibility problems would be those who might benefit from further improvements in planning. However, it is surprising that some individuals showed work improvements following a planning improvement even when their memory was poor. This may relate to CRT adding strategies. Support for this view comes from prospective memory research (McDaniel and Einstein, 2011) which is often required in a work context. It relies on the use of multiple processes, one of which depends on a cue capturing attention and therefore does not rely on a memory search. A strategic approach to planning a set of actions would provide such cues and therefore overcome any memory shortcomings. This probably means that the current CRT provides enough strategic support to overcome difficulties in remembering and therefore removes it as a rate limiting factor. Close inspection of the data indicates that moderated mediation may be disproportionately affected by a few individuals and that this type of mediation is specific to sub-populations with particular cognitive characteristics. Sub-groups will need to be taken into account to provide the best chance of identifying a positive signal for new treatments, especially drug treatment at Phase 2 as well as preventing signal loss at Phase 3.

Even though the planning measure accounts for part of the total CRT effect on work quality, 85% of this total effect size is not accounted for by indirect effects via cognitive improvements. This is not to say that the effect of cognitive improvement is insignificant but that, of course, it is in addition to changes in other variables not accounted for here.

Elsewhere it is suggested that metacognitive processes contribute to the way that cognitive processes are used in everyday life (Wykes and Reeder, 2005; Reeder et al., 2006; Wykes and Spaulding, 2011). Recent reviews of cognitive treatments in traumatic brain injury and stroke also indicate the importance of strategies and metacognition (Cicerone et al., 2011). The therapy in this study was aimed at improving metacognitive knowledge (knowledge of strategies for efficient learning) and metacognitive regulation (ability to implement strategies in real life). These skills were emphasised in the meetings between the participants, therapists and the support worker during therapy. These skills are closely akin to planning abilities. It may be that the improved planning leading to improved work demonstrates participants' use of new metacognitive skills.

Factors that may account for the remaining 85% of the total CRT effect on work include response processing that Wykes et al. (1998) found as a rate limiter on rehabilitation. Those with the poorest response processing when making novel responses were less likely to be living more independently which increases the costs of their care (McCrone et al., 1998; Patel et al., 2010). Other variables that may account for the direct effect are social cognition and social discomfort which can affect work (Bell et al., 2009), social and communication skills which differentiated those with good vocational outcomes from those with poor outcomes (Dickinson et al., 2007) and negative symptoms (Greenwood et al., 2005; Bowie et al., 2010) which contribute to the effects of cognition on real world outcome. Any or all of these measures might have an additional impact. However, few have been investigated in a comprehensive analysis of remediation and functional outcomes and perhaps should be part of the next steps in this research field.

The study has strong internal and external validity in using measures that have been shown to improve in conventional CRT studies as well as an environment that is likely to closely resemble psychiatric service provision around the globe. If specific supported work services had been chosen then probably better work outcomes might have been found but it is not clear that this would have allowed any clearer relationships to emerge between cognitive change and work functioning.

### 4.2 Conclusions

In conclusion, the mediator (planning improvement) was able to account for a proportion of the total effect on work quality and despite clear changes in cognition (memory and flexibility) these do not necessarily drive key changes in outcome. This suggests that the choice of remediation programme should not be based solely on its ability to improve cognition but on how cognitive improvement is translated into functional outcome. We hope that this type of analysis will stimulate others to carry out similar model testing to inform the cognitive remediation of the future. More than one thousand people have received cognitive remediation to date and this corpus of data already provides an opportunity to explore possible underlying mechanisms.

## Role of the funding source

The study was supported by the Medical Research Council UK and is subject to their funding agreement to support free access via PMC to the paper six months post publication. The funding source has played no part in the production of this paper and it represents the opinions solely of the authors.

## Contributors

Til Wykes and Clare Reeder designed the study and wrote the protocol. Sabine Landau managed and under took the statistical analyses with Til Wykes. Til Wykes wrote the first draft of the manuscript and all authors contributed to and have approved the final manuscript.

## Conflict of interest

No authors have any conflicts of interest to declare.

## Figures and Tables

**Table 1:** Demographic and clinical characteristics.

Summaries of baseline measures			
	N	Mean (median) [number]	s.d. (min to max)
*Demographic and other information*			
Age (years)	43	40.5	10.3
No. diagnosed schizophrenia	43	93%	
Gender (number women)	43	[12]	
Ethnicity (number white British)	43	[23]	
Living (number living alone)	43	[15]	
Education (number at higher level)	43	[11]	
Length of education (years)	43	13.1	2.1
Premorbid IQ	43	97	10.3
Current IQ	43	91.9	17.6

*Clinical information*			
PANSS symptoms	42	49.0	11.2
Rosenberg self-esteem	43	22.3	5.1
HAD score	43	15.0	8.2
General functioning score	43	67.4	8.8
First admitted more than 5 years ago	43	[27]	
Mean no. of CRT sessions attended	43	30.2	4.6

**Table 2:** Predicted change over time in cognitive and work measures.

	Predicted mean score (SE)			Test of time 2 versus time 1 [standardised effect size, 95% CI]	Test of time 3 versus time 1 [standardised effect size, 95% CI]
	Time 1	Time 2	Time 3		
*Cognitive outcomes*					
Memory WAIS-III digits total score	14.7 (0.54)	15.8 (0.55)	15.4 (0.56)	z = 2.14, p = 0.032 [d = 0.34, 0.033 to 0.65]	z = 1.41, p = 0.16 [d = 0.23, − 0.09 to 0.55]
Cognitive flexibility WCST total number of errors	59 (3.8)	50 (3.8)	50 (3.8)	z = − 3.77, p = < 0.001 [d = − 0.35, − 0.53 to − 0.17]	z = − 3.68, p < 0.001 [d = − 0.35, − 0.54 to − 0.17]
Planning log-odds zoo map score	4.02 (1.40)	4.45 (1.50)	4.59 (1.51)	z = 1.30, p = 0.10 [OR = 1.54, 0.80 to 2.94]	z = 1.76, p = 0.079 [OR = 1.76, 0.94 to 3.32]

*Work outcomes*					
Square root structured occupation hours	3.48 (0.21)	3.50 (0.22)	3.41 (0.22)	z = 0.20, p = 0.84 [d = 0.01, − 0.13 to 0.16]	z = − 0.57, p = 0.57 [d = − 0.04, − 0.20 to 0.11]
WBI total score	125.0 (3.18)	136.4 (3.52)	138.3 (3.78)	z = 3.24, p = 0.001 [d = 0.46, 0.18 to 0.74]	z = 3.51, p < 0.001 [d = 0.53, 0.24 to 0.83]
WBI work quality score	24.0 (0.76)	26.3 (0.83)	26.9 (0.88)	z = 3.10, p = 0.002 [d = 0.42, 0.16 to 0.69]	z = 3.59, p < 0.001 [d = 0.52, 0.24 to 0.81]

**Table 3:** Moderation of effects of change in planning (Zoo Map profile score) on work outcomes by cognitive flexibility (WCST errors) or memory (Digit span).

	Effect estimate (p-value in brackets) (change in work outcome per 1 point change in planning score)		
	Square root structured occupation hours	WBI total score	WBI work quality score
*Memory Digit span total*			
Low	− 0.027 (p = 0.85)	7.92 (p = 0.06)	2.17 (p = 0.009)
High	− 0.047 (p = 0.80)	2.01 (p = 0.75)	0.16 (p = 0.89)

*Cognitive flexibility WCST total errors*			
Low	− 0.081 (p = 0.57)	6.56 (p = 0.094)	1.84 (p = 0.027)
High	0.061 (p = 0.76)	5.13 (p = 0.32)	1.08 (p = 0.32)
